# An Updated List of Neuromedicinal Plants of Pakistan, Their Uses, and Phytochemistry

**DOI:** 10.1155/2019/6191505

**Published:** 2019-03-03

**Authors:** Abdul Waheed Khan, Arif-ullah Khan, Syed Muhammad Mukarram Shah, Aziz Ullah, Muhammad Faheem, Muhammad Saleem

**Affiliations:** ^1^Department of Pharmacy, University of Lahore, Islamabad, Pakistan; ^2^Riphah Institute of Pharmaceutical Sciences, Riphah International University, Islamabad, Pakistan; ^3^Department of Pharmacy, University of Swabi, Khyber Pakhtunkhwa, Pakistan; ^4^Department of Pharmacy, Forman Christian College, Lahore, Pakistan

## Abstract

**Background:**

Almost every region of Pakistan is stacked with a large number of medicinal plants. Due to high cost and unavailability of allopathic medicines for the neurological diseases, especially in rural areas, traditional healers prescribe phytotherapy for various neurological diseases like epilepsy, depression, anxiety, insomnia, Alzheimer, and migraine. Such treatments are considered to be most effective by the native people.

**Methods:**

The data was collected from articles published on medicinal plants of various districts of Pakistan, using article search engines like Medline, Pubmed, Web of Science, Science Direct, and Google Scholar. Also, information regarding various neurological uses and mode of applications of medicinal plants was obtained from traditional healers, folk medicine users, and local elderly people having knowledge of medicinal plants.

**Results:**

A total of 54 families were found to be used in various neurological diseases, of which the highest use was of Solanaceae (22.22%), Asteraceae (12.96%), Lamiaceae, Papaveraceae, and Poaceae, 9% each, and Caprifoliaceae, Cucurbitaceae, Rhamnaceae, and Rosaceae, 5.5% each. According to districts, 15% of plants that were effective in neurological affections were found in Bahawalpur, 11% in Swat, 8% in Muzaffarabad, 7% in Malakand, and 6% in Bahawalnagar, Dir, Gilgat, and Sarghoda each, with 5% in Dera ghazi khan and Jhelum each. According to the plant's habit, out of total of 103 plants, 61.15% were found to be herbs, 22.33% trees, 11.65% shrubs, and 4.85% climbers. According to the part used of plant, whole plant, leaves, fruits, roots, seeds, and flowers were found to be used 32.03%, 24.27%, 20.38%, 16.50%, 13.59%, and 11.65%, respectively. According to disease's types, 45.63% were found to be effective in insomnia, 31.06% in epilepsy 12.62% in depression, 6.80% in anxiety, 7.77% in hysteria, and 5.88% in migraine.

**Conclusion:**

Taking into consideration this useful knowledge on medicinal properties of the plants for curing neurologic diseases, it is believed that research in areas of ethnomedicine and ethnopharmacology can bring auspicious results that have potential of adding value to the very rich natural resources of Pakistan. This study will help all the researchers from diverse backgrounds working on plants based medicine for neurological diseases.

## 1. Introduction

Globally, neurological diseases are among the major contributors to mortality and morbidity, particularly in developing nations. The well-known manifestations of neurological diseases include mood swing, restlessness, hopelessness, poor coordination, seizures, impaired cognition, paralysis, distress of sensation, muscle weakness, pain, and confusion [1]. There are more than six hundred neurological diseases, some of which are relatively common and well known while others are rare or poorly recognized [2]. Demographic, socioeconomic, and geographic conditions are the major factors affecting epidemiology of neurological diseases. Globally, the overall burden of neurological diseases is about 6.5%. In lower income countries, neurological diseases range from 4 to 5%, as compared to high income countries where such diseases range from 10 to 11%. This high ratio of neurological diseases in advanced countries may be due to their more advanced public health system and health-related facilities that provide and maintain complete data of their patients [1].

About 45 million people of the world, above 18 years of age, suffer from schizophrenia at some stage of their lives, 340 million are affected by depression, and both these diseases are accountable for 60 % of all suicides, while Alzheimer and epilepsy affect about 11 and 45 million people, respectively, around the world accounting for 1% of the total disease burden in the world [3].

In Pakistan, about 10 % people suffer from mental diseases, representing a foggy picture with 2% prevalence of' epilepsy, 5% depression, 1% Alzheimer, and 1.5% schizophrenia [4] as shown in (Table 1). These mental morbidities are responsible for high suicidal rate. Major factors contributing to this alarming increase in mental diseases are unemployment, poverty, political unreliability, violence, and other social horrors and evils beyond the genetic and biological susceptibility [5].

Medicinal plants have been used from the very beginning in health care systems. Studies have been carried out globally to verify their efficacy and some of the findings have led to the production of plant-based medicines. Due to limited access to modern medicine, the local population uses medicinal plants to treat most diseases [6, 7]. Recent focus on plant research has increased worldwide and most evidence has been collected to determine the immense potential of medicinal plants [8]. Medical plants have therapeutic benefits and fewer side effects in comparison with synthetic drugs [9]. Drugs used for neurological diseases along with their side effects are given in (Table 2).

Herbs may provide a source of new compounds including many drugs that are derived from plant sources. For several neurological diseases, modern medicine offers symptomatic treatment that is often expensive and associated with side effects. Indian system of medicine has traditionally been used in several neurological conditions. The accessibility, cost effectiveness, and lower incidence of side effects of plant products offer considerable advantages [10].

Various plant extracts have been screened and investigated for their potential neuropharmacological activities in different experimental models of animals comprising mice and rats. Herbal extracts and natural products including* Bacopa monnieri, Cannabis sativa*,* Solanum nigrum, Withania somnifera, Papaver somniferum*, Zizyphus jujube,* Tribulus terrestris,* and* Verbena officinalis* showed different neuropharmacological activities. These agents can be used alone or as adjuncts to standard drugs, used for various neurological diseases like depression, epilepsy, schizophrenia, Alzheimer, Parkinson, hysteria, melancholia, and dementia, for increasing their efficacy and decreasing side effects.

In developing countries, plant-based medicines are being used by 75-80% of population [11]. The knowledge of indigenous medicinal plants is a part of Pakistani culture and traditionally, majority of Pakistani people use herbal medicines for various diseases [12].

In Pakistan, folk medicines have more use in rural and less developed areas for the treatment of various diseases because of easy access, cost effectiveness, less side effects, and unavailability of allopathic therapeutic agents [13]. This type of treatment, using traditional medicinal flora, is practiced regularly in homes and transferred from generation to generation as a cultural virtue. However, this tradition and associated knowledge are diminishing rapidly due to negligence and less interest of new generation to receive this gift of ethnomedicinal prosperity from their ancestors. Various parameters like industrialization, migration from rural to urban areas for education and jobs, passion towards advanced lifestyles, deforestation, and allopathic medicine might have brought this change in behavior. Therefore, before it is lost forever, this valuable traditional knowledges need to be urgently collected and systematically documented for the interest of humanity [14].

## 2. Materials and Methods

First the articles published on the medicinal plants of various districts of Pakistan were searched in online research database, i.e., Medline, PubMed, Web of Science, Science Direct, and Google Scholar, by using special key words “medicinal plants”, herbal plants, neurological diseases, specific districts names, antialzheimer, antiparkinson, antidepression, sedative, anxiolytic, antiepileptics, epidemiology, and prevalence, from January to March 2018, and downloaded. These entire articles were then viewed and the data of medicinal plants, which have neurological effects, were collected and tabulated in (Table 3). We have personally visited districts Bahawalpur, Bannu, Buner, Dir, Gilgat, Islamabad, Jhelum, Malakand, Mianwali, Rawalpindi, Sargodha, and Swat in April-June 2018 and collected information regarding plants local names, local use, mode of applications, and administration of these plants in neurological diseases from local traditional healers, folk medicine users, and local elderly people of those districts having knowledge of medicinal plants. Information was also collected from distant districts with the help of friends living there via social media (phone calls, text messages, WhatsApp calls and messages, and emails).

## 3. Results and Discussion

**Figure 1:**
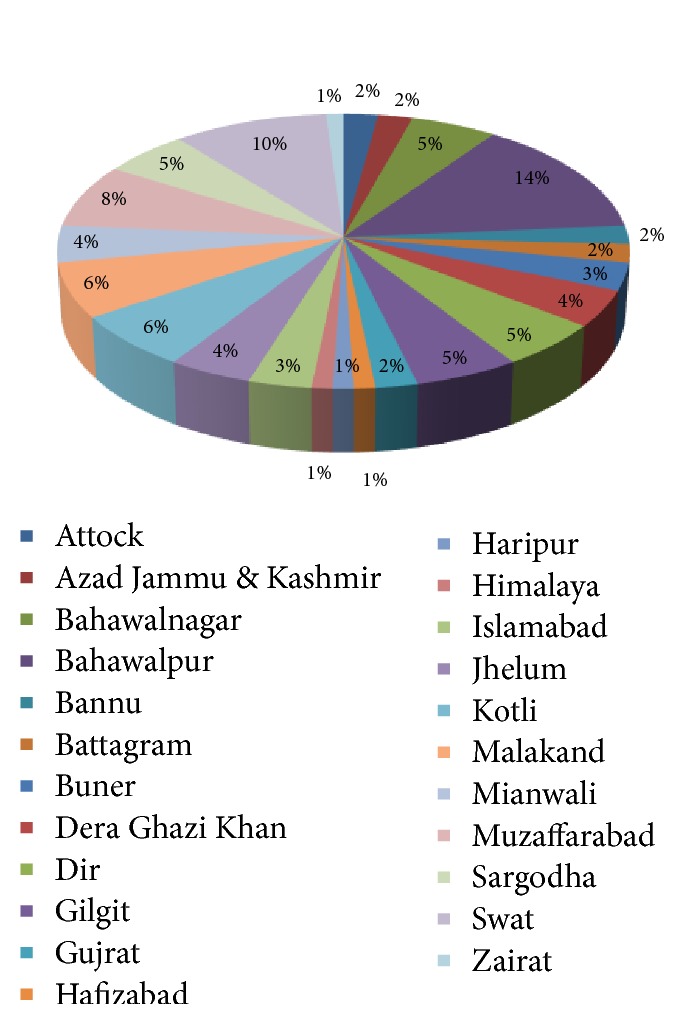
District-wise percentage of plants used for neurological diseases.

A total of 54 families were found to be useful in various neurological diseases, of which the highest use was of Solanaceae (22.22 %), Asteraceae (12.96 %), Lamiaceae, Papaveraceae, and Poaceae, 9 % each, and Caprifoliaceae, Cucurbitaceae, Rhamnaceae, and Rosaceae, 5.5 % each (Table 3). As per district point of view, 15% plants, effective in neurological affections, were found in Bahawalpur, 11% in Swat, 8 % in Muzaffraabad, 7% in Malakand, and 6% in Bahawalnagar, Dir, Gilgat, and Sarghoda each, with 5% in Dera ghazi khan and Jhelum each (Figure 1).

**Figure 2:**
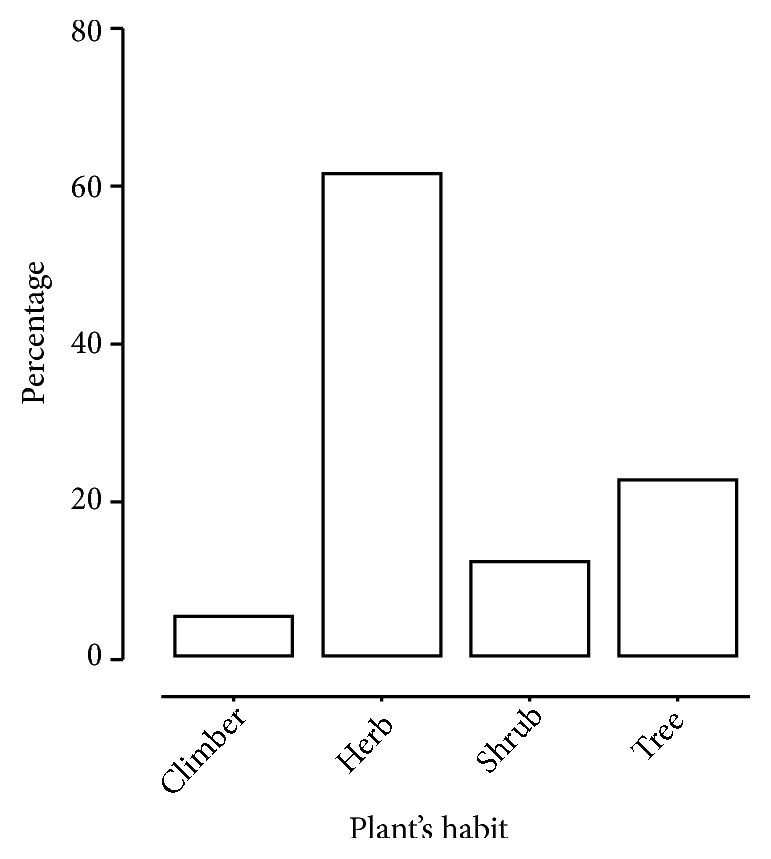
Habit-wise percentage of plants used for neurological diseases.

This district-wise plant distribution will help the researchers, who are willing to research in neuropharmacological area, to easily collect the target plants from the regions to which the plants belong. According to the plant's habit, out of total of 103 plants, 61.15% were found to be herbs, 22.33 % trees, 11.65% shrubs, and 4.85% climbers (Figure 2).

**Figure 3:**
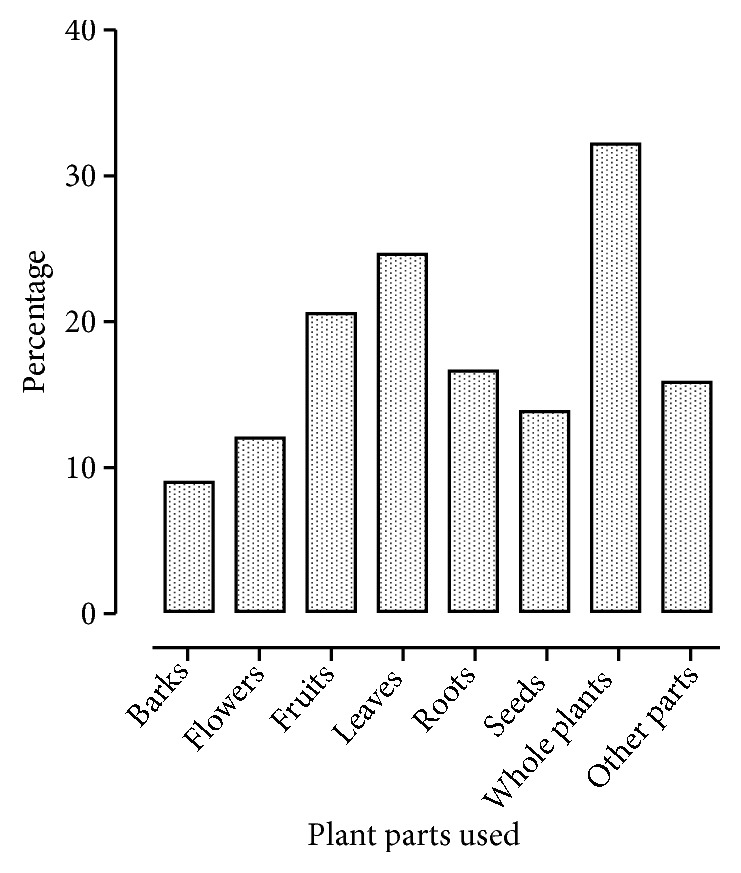
Parts-wise percentage of plants used for neurological diseases.

The habit of plants shows that herbs are most important according to neuropharmacological point of view which is another benefit for researchers working in neuropharmacological area to concentrate on herbs more while selecting neurological active plants. According to the part used of plant, whole plant, leaves, fruits, roots, seeds, flowers, and other parts (bulbs, latex, gum, tubers, and rhizome) were found to be used 32.03 %, 24.27 %, 20.38 %, 16.50 %, 13.59 %, 11.65 %, and 15.53 %, respectively (Figure 3). As some plants have more than one part to be used for various neurological diseases, so such plants were counted into percentage of all respective parts. This division of neuropharmacological plants ensures the researchers to select the most appropriate parts of plants having specific neuropharmacological activities, for their research, as used by traditional healers and folk medicine users.

**Figure 4:**
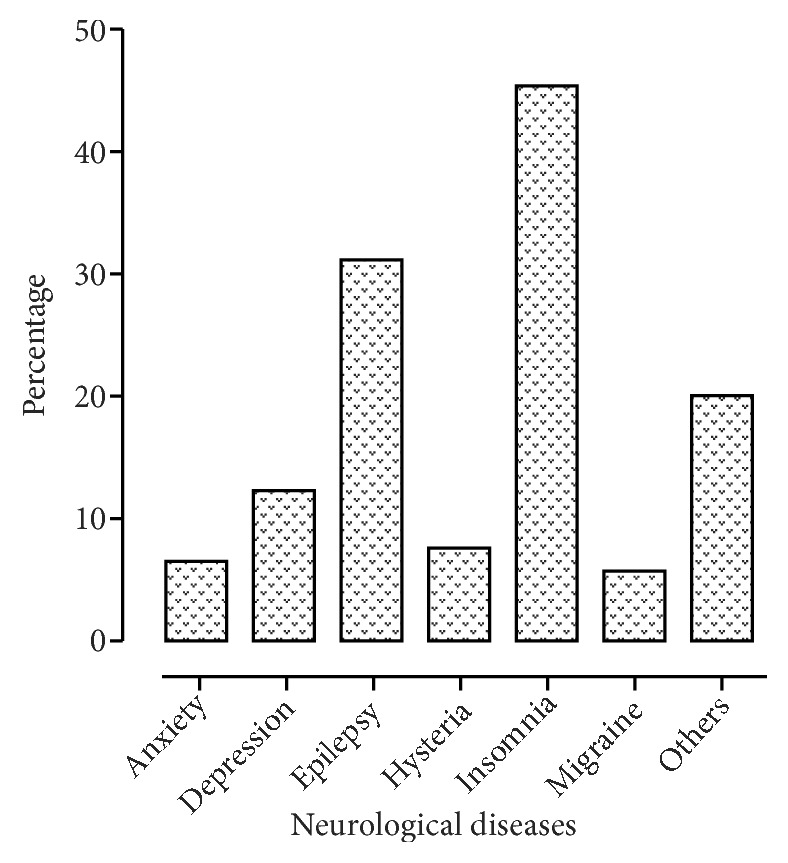
Disease-wise percentage of plants used for neurological diseases.

According to disease's types, 45.63 % were found to be of therapeutic value in insomnia, epilepsy (31.06%), depression (12.62%), anxiety (6.80%), hysteria (7.77%), and migraine (5.88%) and 20.38 % in other neurological diseases (neuralgia, mania, Parkinson, schizophrenia, and nerve pain) (Figure 4). As some plants are used for multiple neurological ailments, so such plants were counted into percentage of all respective diseases. This disease-wise plant division will help the local researchers to select their interest areas in the field of neuropharmacology, by selecting the neurological disease, for which most of the plant's percentage was found to be used by traditional healers and folk medicine users in various districts of Pakistan.

The pharmacological activities of plants are due to the presence of various phytochemicals mainly alkaloids, flavonoids, tannins, saponins, resins, glycosides, terpenoids, phenols, sterols, essential oils, vitamins, and nutrients. Some of these are effective in the treatment of neurological diseases; some are useful for cardiovascular, respiratory, and gastrointestinal diseases while others have chemotherapeutic and antibacterial effects. Some of the important phytochemicals of the plants (Table 4) including alkaloids (like nicotine and scopolamine) are reported to have anxiolytic, antidepressant, and anti-Parkinson activities [15–18], saponins (like bacosides) have been reported for anxiolytic, antiepileptic, antiamnesia, and neuroprotective and memory enhancement activities [19–22], terpenoids (like cannabigerol, tetrahydrocannabinol, and cannabidiol) are reported for their neuroprotective effects [23], flavonoids (like kaempferol, luteolin, quercetin, rutin, and hesperidin) have been reported for their anxiolytic, antidepressant, antiepileptic, anti-Alzheimer, and neuroprotective and memory enhancement activities [24–30], glycosides (like hastatoside and verbenalin) are reported for sleep promoting activity [31], steroids (like sitoindosides VII–X and withaferin-A) have been reported for anxiolytic activity [32].

*Bacopa monnieri *plant is reported for anxiety, depressant, epilepsy, and Parkinsonism and contains alkaloids (Brahmin, nicotine, herpestine, and bacosides A & B), saponins (hersaponin and monnierin), flavonoids (luteolin and apigenin), and sterols like b-sitosterol and stigma-sterol. These constituents are already reported for such neuropharmacological properties and so might be responsible for said activities of this plant [33–36].

*Cannabis sativa* L. has been reported for the treatment of depression, anxiety, convulsion, Alzheimer, dementia, and insomnia and its constituents responsible for these properties are cannabigerol, tetrahydrocannabinol, and cannabidiol [37–41].

*Verbena officinalis *Linn. has been reported as anxiolytic, antidepressant, anticonvulsant, and sedative and its constituents responsible for these activities are verbenin, verbenalin, hastatoside, kaempferol, luteolin, verbascoside, aucubin, and apigenin [42–44].

*Withania somnifera* has been shown to have anxiolytic, antidepressant, anticonvulsant, and anti-Parkinson effects, mainly due to the presence of withanolides, sitoindosides VII–X, and withaferin-A [45–48].

These chemical constituents of plants act on the central nervous system through various mechanisms including regulation of neurotransmitters like adrenergic, cholinergic and serotonergic activity, acting through receptor like GABA and N-methyl-D-aspartate, and ion channels like sodium, potassium, and calcium ion channels. Some of the plant-based drugs and phytochemicals which either are approved or are under clinical trials for the treatment of neurological diseases, mechanism of actions, and their current status in clinical trials are given in (Table 5).

Taking into consideration this useful knowledge on the medicinal properties of plants for curing neurologic diseases, it is believed that the research in the areas of ethnomedicine and ethnopharmacology can bring auspicious results that have potential of adding importance to the very rich natural resources of Pakistan. Various phytochemicals from the above medicinal plants can be further researched under clinical trials and better drugs for treatment of neurological diseases can be obtained with outstanding results and lesser side effects. This study will help all the researchers, especially from Asian countries including Pakistan, China, Iran, India, Sri Lanka, and Bangladesh, working on plants based medicine for neurological diseases.

## 4. Conclusion

The mental illnesses are one of the major problems of the world mainly in communities presenting with poor socioeconomic conditions. In Pakistan and other countries of this region, there is no accurate and up to date record of the neurological ailments. In order to find any treatment for these diseases, first realistic survey would be required to find out the exact percentage of various neurological diseases. Being an alarming psychiatric problem, Alzheimer opens a new area of research, affecting an enormous part of world population, but it is still untreatable. A lot of attempts have been conducted but still there is no such drug that can either slow or stop the process of Alzheimer disease. Allopathic medicines are available for psychological diseases including anxiety, depression, epilepsy, Parkison, and Alzheimer, but these are either not so effective or costly or have serious associated adverse effects. The world is full of natural medicinal resources, of which the main source is plant. We should invest money and go for systemic scientific investigations to perceive such drug candidates' form these plants, which are most efficacious, have minor side effects, and are cost friendly. For this purpose, this study is a gift for researchers who have interest to design and perform research based activities in the field of neuropharmacology by evaluating the unexplored medicinal plants mentioned here for their folkloric uses, determining its mechanistic pathways and identifying chemical constituents responsible for therapeutic effects.

## Figures and Tables

**Table 1:** Global epidemiology of neurological diseases and theircomparative prevalence in Pakistan and neighboring countries.

	Migraine	Stroke	Epilepsy	Depression	Anxiety	Parkinson	Alzheimer
Worldwide	14.9% [49]	5% [50]	0.5-1% [51]	4.4% [52]	3.6% [52]	1% [53]	11.2% [54]
Asia	9.1% [55]	0.94%[56]	0.49% [57]	4.4% [52]	2.8% [58]	0.63% [59]	1.9% [54]
Africa	5.61% [60]	0.4% [61]	1.13% [62]	5.2% [52]	4.4% [52]	0.44% [63]	1.6% [54]
North America	14.4% [64]	2.7% [65]	0.8% [66]	10.6% [67]	7.7% [58]	1.3% [59]	6.4% [54]
South America	11.6% [64]	0.7% [68]	0.98% [57]	13.8% [67]	10.4% [69]	2.3% [59]	4.6% [54]
Europe	15% [70]	6.25%[71]	0.82% [62]	4.2% [52]	3.9% [52]	1.6% [72]	4.4% [54]
Australia	6% [73]	1.8% [74]	0.44% [75]	5.9% [52]	7% [52]	0.46% [76]	6.4% [77]
Pakistan	26.1% [78]	0.25% [79]	2% [4]	4.2% [52]	3.5% [52]	0.23% [51]	1% [4]
India	25.2% [51]	3.69%[71]	0.39% [80]	4.5% [52]	3.0% [52]	0.07% [81]	1.91% [82]
Iran	14% [51]	0.36% [51]	1.8% [80]	4.9% [52]	4.6% [52]	0.29% [83]	2.3% [84]
China	9.3% [78]	4.3% [71]	0.3% [80]	4.2% [52]	3.1% [52]	1.7 % [85]	3.21% [85]
Afghanistan	0.9% [86]	5.2% [87]	8.9% [88]	51.8% [89]	38.5% [89]	35.4% [90]	15.3% [91]

**Table 2:** Side effects of currently using drugs in treatment of various neurological diseases.

Drug Class	Subclasses	Drugs	Side effects	References
Antidepressants	TCA	Imipramine, Amitriptyline, Desipramine, Nortriptyline, Doxepin	weight gain, sedation, dry mouth, nausea, blurred vision, constipation, tachycardia, dry mouth, constipation, hypotension, increased heart rate	[92]
	MAOI	Isocarboxazid, Phenelzine Tranylcypromine, Selegiline	weight gain, fatigue, sexual dysfunction, nausea, hypotension, dry mouth, diarrhea or constipation, headache, drowsiness, insomnia	
	SSRI	Fluoxetine, Paroxetine, Fluvoxamine, Sertraline, Citalopram	headache, sedation, dizziness, nervousness, somnolence, extrapyramidal effects, nausea, dry mouth, diarrhea, agitation, insomnia, sexual dysfunction, weight gain,	[93]
	SNRI	Venlafaxine, Duloxetine, Desvenlafaxine, Levomilnacipran	nausea, insomnia, dry mouth, headache, increased blood pressure, sexual dysfunction, weight gain, urinary retention, hyponatremia, tremors, vertigo, tachycardia, shock-like sensations, paresthesia, myalgia, tinnitus, neuralgia, ataxia	[92, 94]
	Atypical	Bupropion, Mirtazapine, Trazodone, Vilazodone	headache, agitation, insomnia, sweating, sedation, increased appetite, weight gain, nausea, dizziness	[92]

Anxiolytics	BZDs	Alprazolam, Clonazepam, Lorazepam, Midazolam, Diazepam	sedation, memory disturbances, tolerance, fatigue, dependence, drowsiness, lethargy, At higher dosages, impaired motor coordination, dizziness, vertigo, slurred speech, blurry vision, mood swings, euphoria	[95]
	Azapirones	Buspirone, Binospirone, Gepirone, Tandospirone	dizziness, drowsiness, headaches, restlessness, nausea, diarrhea	[96]
	BAR	Phenobarbital, Amobarbital, Secobarbita, Butabarbital, Pentobarbital	sedation, dizziness, headache, nausea, withdrawal include, tremors, agitation, abnormal breathing, coma, confusion, fainting, hallucinations	[97]

Anti-Alzheimer	AChEIs	Donepezil, Rivastigmine, Galantamine	vomiting, diarrhea, weight loss, bradycardia, insomnia, nausea, agitation, syncope	[98]
	Anti-A*β*	Bapineuzumab, Solanezumab, Gantenerumab	microhemorrhage, vasogenic edema, arrhythmia, skin and subcutaneous tissue disorders	
	NMDAR Antagonists	Memantine	psychosis, nausea, vomiting, memory impairment, and neuronal cell death, drowsiness	[99]

Anti-Parkinson	DA	Bromocriptine, Pergolide, Cabergoline, Pramipexole	nausea, hypotension, confusion, delirium, pulmonary fibrosis, vasospasm, erythromelalgia, sleep attacks	[100]
	COMT Inhibitors	Entacapone, Tolcapone	dyskinesia, nausea, confusion, urine discoloration, diarrhea, abdominal pain	
	MAO-B	Selegiline	confusion, delirium, hallucinations, unusual thoughts or behavior, dizziness, nausea, insomnia, trouble breathing	

Antiepileptic	Sodium Channel Blockers	Phenytoin, Carbamazepine, Lamotrigine, Lacosamide, Oxcarbazepine,	dizziness, drowsiness, diplopia, nausea, vomiting, fatigue, ataxia, neurotoxicity, cardiac arrhythmias, hirsutism, hepatotoxicity, steven-johnson syndrome	[101]
	Calcium Channel Blockers	Ethosuximide, Zonisamide, Trimethadione	nausea, vomiting, headache, mental status changes, neuropathy, change in weight	[102]
	GABA transaminase Inhibitors	Vigabatrin, L-Cycloserine, Ethanolamine-O-Sulfate, Valproate	drowsiness, nystagmus, hyperexcitability, insomnia, fever, memory impairment, depression, confusion, agitation, asthenia, laryngitis, weight gain, vomiting	[103]

TCA: tricyclic antidepressant; MAOI: monoamine oxidase inhibitor; SSRI: selective serotonin reuptake inhibitor; SNRI: serotonin norepinephrine reuptake inhibitor; BZDs: benzodiazepines; BAR: barbiturates; AChEIs: acetylcholinesterase inhibitors; A*β*: amyloid beta; NMDAR: N-methyl-D-aspartate receptor; DA: dopamine agonists; COMT: catechol-O-methyltransferase; MAO-B: monoamine oxidase B; GABA: gamma-aminobutyric acid.

**Table 3:** Traditionally used medicinal plants for the treatment of various neurological diseases.

S #	Botanical Name	Local Name	Family	Habitat	Part Used	Used for	Mode of Applications	Location	Reference
1	*Achyranthes aspera*	Ayokanda	Amaranthaceae	Herb	Leaves and Shoot	Nerve tonic	Paste of dried leaves and shoots is applied on head	Sargodha	[104]

2	*Ailanthus altissima*	Backyanra	Simarubacea	Tree	Bark	Hysteria	Decoction of bark to make tea	Malakand	[105]

3	*Albizia lebbeck*	Sirin	Mimosaceae	Tree	Roots	Depression, Migraine and Anxiety	Decoction of root to make tea	Mianwali	[106]

4	*Allium sativum *	Ooga	Amaryllidaceae	Herb	Bulbs and Leaves	Hysteria and Epilepsy	Decoction of bulbs and leaves	Swat	[107]

5	*Alnus nitida *	Geiray	Betulaceae	Tree	Flowers	Insomnia	Powder of dried flowers mixed with water and used orally	Dir	[108]

6	*Alternanthera sessilis*	Waglon	Amaranthaceae	Herb	Leaves	Neuralgia and Sedative	Sniffing of leaves sap	Bahawalpur	[109]

7	*Anagallis arvensis*	Billy booti	Primulaceae	Herb	Whole plant	Nervine, mania and Epilepsy	Extract of whole plant	Bahawalpur	[109]

8	*Artemisia scoparia *	Jaukay	Asteraceae	Herb	Roots	Epilepsy	Powder of roots taken with water	Dir	[108]

9	*Asparagus officinalis*	Phala-moosa	Asparagaceae	Herb	Leaves	Insomnia	Tea of leaves are used on empty stomach	Lahore	[110]

10	*Atropa accuminata*	Bargak	Solanaceae	Herb	Leaves	Insomnia and narcotic	Powder of leaves are taken with water	Dir	[108]

11	Avena fatua	Jodal	Poaceae	Herb	Seeds	Depression and nervous exhaustion	Either the seeds fluid extract or oatmeal obtained by crushing and grinding seeds	Dera Ghazi Khan	[111]

12	*Avena sativa*	Jai	Poaceae	Herb	Seeds	Nerve tonic and Insomnia	A tincture of juice of immature seeds	Islamabad	[112]

13	*Bacopa monnieri *	Brahmi sak	Scrophulariaceae	Herb	Whole plant	Epilepsy	Extract of whole plant is taken orally	Mianwali	[106]

14	*Buglossoides arvensis*	Kalu	Boraginaceae	Herb	Leaves	Insomnia	Infusion of leaves is used orally	Kotli	[113]

15	*Caltha alba*	Makanpat	Ranunculaceae	Herb	Whole plant	Insomnia	Extract of whole plant	Dir	[108]

16	*Campanula pallida*	Beli Flower	Campanulaceae	Herb	Flowers	Insomnia	An infusion of flowers is used orally	Kotli	[113]

17	*Cannabis Sativa*	Bhang	Cannabaceae	Herb	Flowers	Insomnia	The ground flowers are used by mixing with other fruits	Bannu	[114]

18	*Capparis decidua*	kdler	Capparidaceae	Shrub	Flowers, fruits and shoots	Insomnia	The powder of flowers and shoots while fruits are eaten as such	Gawadar	[115]

19	*Capparis spinosa *	Kawir	Capparidaceae	Shrub	Whole plant	Mental disorders	Fresh extract of whole plant is used	Gilgat	[116]

20	*Carthamus tinctorius *	Tukhmiga-rtum	Asteraceae	Herb	Roots, oil and flowers	Insomnia	Decoction of roots to make tea while oil is applied externally	Rawalpindi	[117]

21	*Celtis australis *	Karr	Cannabaceae	Tree	Bark	Epilepsy	Decoction of bark is used orally	Sargodha	[118]

22	*Cenchrus pennisetiformis*	Cheetah- gha	Poaceae	Herb	Leaves and fruits	Epilepsy	Extracts and juice of leaves and fruits	Hafizabad	[119]

23	*Citrullus colocynthis*	Tumma	Cucurbitaceae	Climber	Roots and fruits	Epilepsy	The extract of roots is taken with water while fruit's powder is mixed with sugar	Jhelum	[120]

24	*Citrus limon*	Nimboo	Rutaceae	Tree	Whole plant	Anxiety and Depression	whole plant extract	Bahawalpur	[109]

25	*Citrus medica*	Khatti	Rutaceae	Tree	Leaves, seeds, latex	Insomnia	Powder of leaves, seeds and dry latex are taken orally with water	Bahawalpur	[109]

26	*Colebrookia oppositifolia*	Lansa	Lamiaceae	Shrub	Leaves and roots	Epilepsy	Fresh leaves extract and roots decoction tea is taken orally	Malakand	[121]

27	*Commiphora wightii *	Guggul, Mukul	Burseraceae	Herb	Gum	Nervous diseases	The aqueous extract of gum is used	Muzaffarabad	[122]

28	*Convolvulus arvensis*	Baily	Convolvulaceae	Herb	Whole plant	Epilepsy	whole plant extract	Malakand	[121]

29	*Cucurbita maxima *	Walayti kadoo	Cucurbitaceae	Climber	Fruits	Nervous disorders	Juice of both unripe and ripe fruits is used	Azad Jammu & Kashmir	[123]

30	*Cuscuta reflexa *	Bepari, Kasus	Cuscutaceae	Tree	Seeds	Insomnia	An infusion of seed is used	Muzaffarabad	[122]

31	*Cymbopogon citratus*	Lemon- grass	Poaceae	Herb	Oil of whole plant	Nervous system tonic	Oil is externally applied on head	Bahawalpur	[109]

32	*Cynodon dactylon*	Lawn grass	Poaceae	Herb	Whole plant	Epilepsy and Hysteria	Extracted juice of plant is used	Dera Ghazi Khan	[111]

33	*Cyperus rotundus*	Deela	Cyperaceae	Herb	Tubers	Epilepsy	Oil obtained from tubers are used	Bahawalnagar	[124]

34	*Datura alba*	Datura	Solanaceae	Shrub	Leaves and seeds	Neuralgia, Epilepsy, Hysteria and Insomnia	Lotion of seed's powder is applied locally for neuralgia while tea of leaves is used for Epilepsy	Bahawalpur	[109]

35	*Datura innoxia *	Datura	Solanaceae	Herb	Leaves	Epilepsy and Insomnia	Extract of leaves in water	Dir	[108]

36	*Datura metel*	Dhaturo	Solanaceae	Herb	Leaves and seeds	Epilepsy and Insomnia	Leaves extract and seed's decoction are used	Muzaffarabad	[122]

37	*Datura stramonium *	Datura	Solanaceae	Herb	Whole plant	Insomnia and Parkinson	Extraction of whole plant is used	Dera Ghazi Khan	[111]

38	*Daucus carota *	Gajar	Apiaceae	Herb	Whole plant	Nerve tonic	Eaten as a whole or its juice is used	Sargodha	[104]

39	*Eclipta alba*	Bhringaraj	Asteraceae	Herb	Roots, oil and leaves	Insomnia	Oil is externally applied while roots and leaves extract is used orally	Bahawalpur	[109]

40	*Eruca sativa *	Tara meera	Cruciferaceae	Herb	Whole plant	Epilepsy	Fluid extraction of plant is used	Islamabad	[112]

41	*Evolvulus alsinoides *	Sankha-holi	Convolvulaceae	Herb	Whole plant	Epilepsy	Decoction of whole plant is used	Islamabad	[112]

42	*Ficus lyrata*	Beeri patta	Moraceae	Tree	Leaves	Migraine	Extraction of leaves is used orally	Bahawalpur	[109]

43	*Flueggea leucopyrus*	Shina	Phyllanthaceae	Shrub	Roots	Epilepsy	Decoction and extraction of roots are used	Dir	[125]

44	*Fumaria indica*	Pitpapra	Fumariaceae	Herb	Leaves and stem	Insomnia	Fresh juice of leaves and stem is used	Rawalpindi	[117]

45	*Gmelina arborea*	Kumbar	Lamiaceae	Tree	Roots	Epilepsy	Extraction and decoction of roots tea is used	Sargodha	[118]

46	*Hyoscyamus niger *	Ajwain-i- Khurasani	Solanaceae	Herb	Leaves and seeds	Insomnia and Nervous afflection	Extraction of fresh leaves and powder of seeds are used orally	Gilgat	[126]

47	*Hypericum perforatum*	Bulhsana	Hypericaceae	Herb	Whole plant	Depression and Insomnia	Fresh extract of whole plant is used orally	Gujrat	[127]

48	*Hyssopus officinalis *	Zufa, Zupa	Lamiaceae	Herb	Whole plant	Nervous affection	Extraction of fresh whole plant	Ziarat	[128]

49	*Indigofera heterantha*	Kainthi	Papilionaceae	Shrub	Whole plant	Epilepsy and neuropathy	Extract of whole plant is used	Gilgat	[116]

50	*Jasminum grandiflorum*	Chanbeli	Oleaceae	Climber	Whole plant	Anxiety, tension and Depression	Oil or tea of leaves and flowers extract are used	Bahawalpur	[109]

51	*Jasminum officinale *	Chanbeli	Oleaceae	Climber	Whole plant	Insomnia	Oil is rubbed on heart as nerve sedative	Swat	[107]

52	*Juglans regia*	Ghuz	Juglandaceae	Tree	Fruits	Depression	Fruits are taken as whole orally	Malakand	[105]

53	*Lactuca serriola*	Berham dandi	Asteraceae	Herb	Whole plant	Memory Enhancing	Fresh plant is ground in water along with black pepper	Jhelum	[120]

54	*Linum usitatissimum*	Alsi	Linaceae	Herb	Stem	Depression, Schizophrenia and Anxiety	Extraction of fresh stem is used	Kotli	[113]

55	*Lycopersicon esculentum *	Tamator	Solanaceae	Herb	Fruits	Nervous weakness	Eaten as a whole or its juice is used	Sargodha	[104]

56	*Martricaria chamomilla*	Babuna	Asteraceae	Herb	Whole plant	Insomnia	Extraction of whole plant is used orally and oil massage or aromatherapy into skin of head is performed	Rawalpindi	[117]

57	*Martynia annua.*	Bichhu-butti	Martyniaceae	Herb	Leaves and fruits	Epilepsy	Juice of leaves or leaves are cooked to make curry and fruits are taken as dry powder with water	Kotli	[113]

58	*Melia azedarach*	Bakyana	Meliaceae	Tree	Leaves	Hysteria	Decoction of leaves to makes tea	Malakand	[105]

59	*Mimordica dioca*	Jungli karela	Cucurbitaceae	Climber	Fruits and seeds	Insomnia	Fruit's extract and seed oil are used	Mianwali	[129]

60	*Moringa oleifera*	Sohan-jana	Moringaceae	Tree	Seeds and bark	Migraine	Seeds oil used externally while powder of leaves	Gujrat	[127]

61	Ocimum basilicum	Niazbo	Lamiaceae	Herb	Leaves, flowers, seeds and roots	Migraine, Insomnia and Depression	Juice of fresh leaves and flowers while oil of seeds is applied externally on head	Bahawalnagar	[124]

62	*Paeonia emodi*	Mamaikh	Paeoniaceae	Herb	Rhizome	Epilepsy	Rhizome powder is given 1/2 teaspoon twice a day	Malakand	[105]

63	*Papaver dubium*	Koko-kanga	Papaveraceae	Herb	Flowers	Insomnia	Fluid extract of flowers is used	Kotli	[113]

64	*Papaver hybridum *	Post	Papaveraceae	Herb	Fruits	Insomnia	Fruit and its decoction are used	Jhelum	[120]

65	*Papaver nudicaule*	Zangali kashkash	Papaveraceae	Herb	Flowers	Insomnia	Fluid extract of flowers is used	Buner	[130]

66	*Papaver rhoeas *	Alak jinai	Papaveraceae	Herb	Flowers	Insomnia	Fluid extract of flowers is used	Buner	[130]

67	*Papaver somniferum *	Qash-Qash	Papaveraceae	Herb	Fruit's latex	Insomnia	Latex of unripe fruit is dissolved in water and used orally	Swat	[107]

68	*Parthenium hysterophorus*	Ragweed	Asteraceae	Herb	Leaves	Insomnia	Leaves extraction is used	Buner	[130]

69	*Peganum harmala*	Harmal	Zygophyllaceae	Herb	Seeds	Hysteria	A small amount of seeds added to sufficient grapes juice, boiled to make thick solution and used orally	Dera Ghazi Khan	[111]

70	*Populus caspica*	Nakhtar	Pinaceae	Tree	Fruits	Insomnia	Whole raw fruits are consumed	Malakand	[105]

71	*Primula veris*	Cowslips	Primulaceae	Herb	Flowers	Insomnia	A tasty wine of flowers is made which is used orally	Gilgat	[126]

72	*Prunus persica*	Ardou	Rosaceae	Tree	Leaves, flowers and fruits	Insomnia	Extract of leaves & flowers and fruits are taken as such	Gilgat	[126]

73	*Punica granatum*	Darrona	Punicaceae	Shrub	Fruits	Memory enhancing	Fruit's juice or fresh seeds are eaten as such	Azad Jammu & Kashmir	[123]

74	*Pyrus communis*	Nashpatai	Rosaceae	Tree	Fruits	Insomnia	Fruits are eaten as such	Dir	[131]

75	*Pyrus pashia*	Tangai	Rosaceae	Herb	Fruits	Insomnia	Fruits are eaten as such	Swat	[107]

76	*Ranunculus muricatus*	Ziar Gulay	Ranunculaceae	Herb	Whole plant	Sciatic and nerve pain	Extraction of dried whole plant is used	Swat	[132]

77	*Raphanus sativus*	Mooli	Brassicaceae	Herb	Seeds	Nervous weakness	Decoction of seeds is used	Sargodha	[104]

78	*Ricinus communis*	Arand	Euphorbiaceae	Shrub	Roots, seeds, leaves	Insomnia and as narcotic	Extract of leaves and roots while oil of seeds are used	Rawalpindi	[117]

79	*Salvadora oleoides*	Peelu	Salvadoraceae	Tree	Whole plant	Epilepsy	Fruit is eaten as raw while tea of leaves and roots are also used	Bahawalpur	[109]

80	*Schinus molle *	False pepper	Anacardiaceae	Tree	Bark and leaves	Depression	Decoction of bark and leaves to make tea	Sargodha	[118]

81	*Scutellaria chamaedrifolia*	Skullcap	Lamiaceae	Herb	Shoots	Insomnia and Depression	Decoction of shoots to make its tea	Swat	[133]

82	*Solanum miniatum*	Peelak	Solanaceae	Herb	Whole plant	Insomnia	whole plant decoction is mixed with sugar	Jhelum	[120]

83	*Solanum nigrum*	Mako	Solanaceae	Herb	Whole plant	Insomnia	Juice of whole plant	Bahawalpur	[109]

84	*Solanum Surratense*	Wara-mara ghinrhye	Solanaceae	Herb	Fruits	Melancholia and Depression	The paste of fruits crushed powders is applied on head externally	Bannu	[114]

85	*Taxus baccata *	Banhya	Taxaceae	Tree	Leaves and fruits	Epilepsy	Extraction of dried leaves and fruits are consumed as such	Swat	[134]

86	*Taxus wallichiana*	Barmi	Taxaceae	Tree	Bark, leaves and fruits	Epilepsy and Insomnia	Extract of dried bark and leaves while flesh of fruits are consumed	Battagram	[135]

87	*Terminalia arjuna*	Arjun	Combretaceae	Tree	Fruits, bark and leaves	Anxiety	Bark infusion left whole night, then its decoction taken early in the morning and used orally	Bahawalpur	[109]

88	*Tribulus terrestris*	Bakhra	Zygophyllaceae	Herb	Whole plant	Epilepsy and Depression	Powder of dried whole plant	Bahawalnagar	[124]

89	*Valeriana jatamansi*	Mushk-bala	Vahliaceae	Herb	Whole plant	Epilepsy and neurosis	Fresh extract of whole plant	Muzaffarabad	[122]

90	*Verbascum thapsus *	Jungle tambako	Scrophulariceae	Herb	Roots	Migraine	Decoction of root to make tea to use as drink	Mianwali	[106]

91	*Verbena officinalis *	Shamkay	Verbenaceae	Herb	Whole plant	Depression, Migraine and Epilepsy	Extract of dried whole plant is used	Battagram	[135]

92	*Viburnum cotinifolium*	Guch	Caprifoliaceae	Shrub	Stem's bark	Insomnia	Extract of stem's bark	Muzaffarabad	[122]

93	*Viburnum opulus*	Sunaira Phul	Caprifoliaceae	Shrub	Bark	Insomnia and Hysteria	Decoction of bark is used	Muzaffarabad	[122]

94	*Viburnum prunifolium*	Blackhaw	Caprifoliaceae	Tree	Root's bark	Hysteria, Anxiety and Epilepsy	Decoction of root's bark is used	Muzaffarabad	[122]

95	*Vicia sativa*	Muttri	Papilionaceae	Herb	Flowers	Epilepsy and nervous disorders	The juice of flowers petals is used	Kotli	[113]

96	*Viola betonicifolia*	Banafsh	Violaceae	Herb	Whole plant	Epilepsy and nervous disorders	Fresh extract of whole plant orally	Malakand	[105]

97	*Viola canescens *	Banafsha	Violaceae	Herb	Whole plant	Insomnia and Epilepsy	Extract and decoction tea of whole plant	Swat	[133]

98	*Withania coagulans*	Paneer doda	Solanaceae	Herb	Fruits, roots and leaves	Nervous Exhaustion, memory loss and tension	Extract of leaves, roots and fruits are used	Bahawalnagar	[124]

99	*Withania somnifera*	Asgandh	Solanaceae	Shrub	Roots	Insomnia	Powder of roots is taken with water	Bahawalpur	[109]

100	*Xanthium strumarium *	Chota dhatura	Asteraceae	Herbs	Fruits, seeds and roots	Insomnia	Decoction of fruits, roots and seeds to make tea	Attock	[136]

101	*Ziziphus jujuba*	Beri	Rhamnaceae	Tree	Leaves, roots and fruits	Anxiety and Insomnia	Extract of leaves, decoction of roots and dried fruits are consumed	Bahawalnagar	[124]

102	*Ziziphus mauritiana*	Ber	Rhamnaceae	Tree	Roots	Nerve tonic	Decoction of roots is used as tea	Sargodha	[104]

103	Ziziphus nummularia	Jangli beri	Rhamnaceae	Shrub	Leaves and fruits	Insomnia	Extract of leaves while fruits are taken as such	Attock	[136]

**Table 4:** Phytochemical constituents and pharmacological properties of some well-known medicinal plants.

S.#	Medicinal Plants	Pharmacological Properties	Part used	Phytochemical Constituents	Chemical Compounds Identified	References
1	*Allium sativum*	1. Antidepressant 2. Anticonvulsant 3. Anti-Alzheimer	1. Dried bulbs 2. Oil 3. Whole garlic	Thiosulfinates, sapogenins phenols, saponins, volatile compounds, antioxidants, flavonoids, vitamins, minerals and proteins	Alliin, allixin, 1,2-vinyldithiin, ajoenes, S-allyl-cysteine sulfoxide, calcium, Potassium, vitamin B and vitamin C	[137–140]

2	*Bacopa monnieri*	1. Antidepressant 2. Anxiolytic 3. Anticonvulsant 4. Anti-Parkinson	1. Leaves 2. Stems and leaves 3. Leaves 4. Conc. tincture of plant	Alkaloid,tannin, saponin, phlobatannin, glycoside, terpenoid, flavonoid, sterols, phenol, steroid, anthraquinone and carbohydrate	Brahmin, nicotine, herpestine, bacosides A & B, hersaponin, betulic acid, monnierin, apigenin, b-sitosterol, stigma-sterol and luteolin	[33–36, 141]

3	Cannabis sativa	1. Antidepressant and anxiolytic 2. Anticonvulsant 3. Anti-Alzheimer and antidementia 4. Sedative	1. Leaves 2. Leaves 3. Flowers 4. Whole plant	Alkaloid, flavonoids, tannins, phenols, resins, cardiac glucosides, terpenes, steroids, volatile oils and balsam	Cannabinoids, cannabidiol, dronabinol, cannabigerol, tetrahydrocannabinolic acid, cannabichromenic acid, cannabidiolic acid, anandamide, cannabigerolic acid and cannabichromene	[37–40, 142, 143]

4	*Hyoscyamus niger*	1. Antidepressant 2. Anti-seizure 3. Anti-Parkinson	1. Leaves 2. Seeds 3. Seeds	Alkaloids, withanolide steroids, lignanamides, tyramine derivative, steroidal saponins, glycosides, lignans, coumarinolignan, and flavonoids	Apoatropine, L-DOPA, Cuscohygrine, choline Daturamine, Hyoscine, tropine, hyoscypicrin, phytin, aphoyoscine, alpha and beeta belladonine and Skimmianine	[144–148]

5	*Solanum nigrum*	1. Anti-seizure 2. Sedative	1. Leaves 2. Fruits	Alkaloids, flavonoids, tannins, saponins, glycosides, proteins, carbohydrates, coumarins and phytosterols	Pinoresinol, syringaresinol, medioresinol, scopoletin, tetracosanoic acid and beta-sitosterol	[149–152]

6	*Withania somnifera*	1. Anti-Parkinson 2. Anxiolytic and antidepressant 3. Anticonvulsant	1. Whole plant 2. Roots 3. Stems and roots	Alkaloids, steroidal lactones, saponins and iron	Withanolides, withaferins, Withanine, isopellertierine, anferine, Anahygrine, Cuscohygrine, Beta-Sisterol, Chlorogenic acid, Scopoletin, choline, Somniferiene, Somniferinine and Tropanol	[45–47, 153]

7	*Papaver somniferum*	1. Anticonvulsant	1. Seeds	Alkaloids, glycosides, tannins, Phytosterols, Terpenoids, Flavanoids and Carbohydrates	Morphine, Codeine, thebaine, noscapine, papaverine, Salutarifine, meconidine, codmine, neoprene, lanthothine, rophyroxine, narcotisline and papaveramine	[154–159]

8	*Ziziphus jujube*	1. Sedative and hypnotic 2. Anxiolytic 3. Anti-seizure	1. Seeds 2. Leaves 3. Fruits	Triterpenic acids, flavonoids, saponins, cerebrosides, amino acids, phenolic acids, vitamins, total sugars and nucleosides	Zizybeoside I and II, Chryseoriol, Swertisin, Quercetin, Jujubasaponin IV, Lotoside I and II, Zizyphus saponin I and II	[160]

9	*Tribulus terrestris*	1. Anxiolytic 2. Antidepressant 3. Sedative	1. Leaves 2. Whole plant 3. Whole plant	Saponins, flavonoids, glycosides, alkaloids and tannins	Tigogenin, neotigogenin, rutin, chlorogenin, caffeoyl, ruscogenin, kaempferol, tribulosid, terrestribisamide, quercetin, *β*-sitosterol, stigmasterols, harmane, norharmane and tribulusterine	[161–164]

10	*Verbena officinalis*	1. Antidepressant 2.Anticonvulsant, anxiolytic and sedative	1. Leaves 2. Whole plant	Alkaloids, flavonoids, diterpenes, proteins, amino acids, tannins, saponins, phytosterols and phenolic compounds	Verbenin, oleanolic acid, verbenalin, hastatoside, alpha-sitosterol, ursolic acid, kaempferol, aucubin, luteolin, verbascoside, apigenin, scutellarein, limonene and spathulenol	[42, 43]

11	*Albizia lebbeck*	1. Anticonvulsant 2. Nootropic and anxiolytic	1. Leaves 2. Leaves	Alkaloids, flavonoids, phenols, saponins; steroids and terpenoids	Albizia saponins A, B and C, albizinin, melacacidin, catechin lebbecacidin, friedelin, and *β*-sitosterol	[165–168]

12	*Avena sativa*	1. Antidepressant 2. Anxiolytic	1. Seeds 2. Whole plant	Carbohydrates, alkaloids, flavanoids, steroids, glycosides, saponins, amino acids, gums and mucilage	Gramine, flavone, apigenin and luteolin, flavonolignans, saponins and ferulic acid	[169–171]

13	*Capparis decidua*	1. Sedative and anticonvulsant	1. Flowers and fruits	Alkaloids, glycosides, terpenoids, sterols, flavanoids, phenols and fatty acids	Capparine, cappariline, capparinine, *β*-sitosterol, capparidisine, capparisine, codonocarpine, Capric acid, cadabacine, quercetin and rutin l-stachydrine	[172, 173]

14	*Citrus limon*	1. Anticonvulsant 2. Sedative, anxiolytic and antidepressant	1. Essential oil of leaves 2. Essential oil of leaves	Phenols, flavonoids, terpenoids, essential oils, carotenoids, citric acid and ascorbic acid	Limonene, *α*-pinene, *β*-pinene, linalool, *α*-terpineol, linalyl acetate, acetate geranyl, nerolidol, acetate neryl, farnesol, sabinene, myrcene, cineol and geranial	[174–176]

15	*Citrullus colocynthis*	1. Anticonvulsant 2. Antidepressant	1. Fruits 2. Fruits	Alkaloids, flavonoids, glycosides, saponosides, Phenolic compounds and ascorbic acid	Colocynthin, colocynthein, colocynthetin, Cucurbitane type triterpen glycoside, quercetin and Flavone	[15, 16, 177]

16	*Datura metel*	1. Antiepileptic 2. Sedative and hypnotics	1. Leaves 2. Seeds	Alkaloids, resins, flavonoids, reducing sugars, tannins, terpenoids and steroid glycosides	Hyoscyamine, scopolamine, atropine, daturabietatriene, daturasterol,, b-sitosterol and Melatonin and serotonin	[17–21]

17	Hypericum perforatum	1. Antidepressant 2. Anti-Parkinson 3. Neuroprotective 4. Anticonvulsant 5. Anti-Alzheimer 6. Anxiolytic and sedative	1. Flowers 2. Flowers and leaves 3. Whole plant 4. Flowers and leaves 5. Flowers 6. Flowers	Phenylpropanes, flavonoids, biflavones, phloroglucinols proanthocyanidins, amino acids, essential oil and naphthodianthrones	Hyperoside, adhyperforin Quercitrin, Rutin, Hypericin, Kaempferol, Biapigenin and Hyperforin	[22–28]

18	*Jasminum grandiflorum*	1. Antidepressant 2. Anticonvulsant	1. Essential oil of plant 2. Leaves	Coumarins, steroids, cardiac glycosides, essential oils, flavonoids, phenolics and saponins	Rutin, kaempferol, quercetin, *β*-primeveroside, kaempferol, hesperidin Methyl jasmonate, methyl anthranilate, linalool *β*-rutinoside, oleuropein and daucosterol	[29–31]

19	*Lycopersicon esculentum*	1. Antidepressant 2. Anticonvulsant 3. Memory enhancement 4. Anti-Parkinson	1. Fruits 2. Dried fruit extract 3. Dried fruit extract 4. Seeds	flavonoids, tannins, saponin, glycosides, Steroids, fatty acids, carbohydrates and proteins	Chlorogenic acid, rutin, naringenin, noradrenaline lycopene, dopamine, tomatin, tomatoside-A, ascorbic acid, bergapten, serotonin and adrenaline	[32, 178–180]

20	Ocimum basilicum	1. Antidepressant 2. Anticonvulsant 3. Anxiolytic and sedative 4. Enhance memory retention	1. Essential oil 2. Leaves 3. Aerial parts 4. Leaves	Terpenoids, essential oil, polyphenols, tannins and flavonoids	Cineole, geraniol, linalool, cadinol and sabinene, methyl chavicol, *β*-caryophyllene and neral, quercetin, myricetin, kaempferol, catechin and eugenol	[181–184]

21	*Punica granatum*	1. Antidepressant 2. Anxiolytic and anticonvulsant 3. Anti-Alzheimer 4. Memory enhancement	1. Fruits 2. Leaves 3. Fruits 4. Fruit's peel	Flavonoids, glycosides, amino acids, pectin, indoleamines, tannins, sterols, polyphenols, carbohydrates, ellagitannins, anthocyanins and triterpenoid	Catechin, rutin, quercetin epicatechin, estriol, luteolin kaempferol, anthocyanins, gallagyldilacton, stigmasterol, *β*-sitosterol, testosterone, tocopherols and isoflavones	[185–188]

**Table 5:** The different phytochemicals effective in various neurological diseases and their current clinical phase status.

Sr #	Phytochemicals	Source	Family	Disease	Mechanism	Development stage	Trade Name	Reference
1	Cannabidiol	Cannabis sativa	Cannabaceae	Epilepsy	Modulation of intracellular calcium and neuronal inhibition	FDA approved, 2018	Epidiolex as 5- 10 mg/kg/day	[189]

2	Cannabidol	Cannabis sativa L.	Cannabaceae	Chronic Neuropathic pain	CB1 and CB2 receptor activation	FDA approved, 2005	Sativex Spray (CBD 25mg/ml + THC27mg/ml)	[190]

3	Capsaicin	Capsicum annum L.	Solanaceae	Postherpetic neuralgia	TRPV1 activator	FDA approved, 2010	Qutenza as Patch (179mg capsacin)	[190]

4	Curcumin	Curcuma longa	Zingiberaceae	Dementia	Anti-amyloid, AChEI	phase II		[191]

5	Galantamine	*Galanthus nivalis*	Amaryllidaceae	Alzheimer	AChEI, allosteric modulation of nicotinic ACh receptor	FDA approved, 2004	Razadyne as 8-12 mg BD	[192]

6	Huperzine A	*Huperzia serrata*	Huperziaceae	Alzheimer	AChEI, inhibits NMDA and glutamate toxicity	approved in China		[193]

7	Ibogaine	Tabernanthe iboga	Apocynaceae	Parkinson	Dopaminergic agonist, NMDA antagonism	preclinical		[193]

8	Psychollatine	Psychotria umbellate	Rubiaceae	Parkinson	MAO inhibitor	preclinical		[193]

9	Resveratrol	Vitis vinifera L.	Vitaceae	Alzheimer	Reduces A*β* formation and promote A*β* decomposition	phase II		[194]

10	Scyllo-Inositol	Cornus florida L.	Cornaceae	Alzheimer	Breakdown of neurotoxic fibrils, allowing amyloid peptides to clear the body rather than form amyloid plaques	phase II		[195]

FDA: food and drug administration; TRPV1: transient receptor potential vanilloid 1; CB1 and CB2: cannabinoid receptor type 1 & type 2; Ach: acetylcholine; AChEI: acetylcholinesterase inhibitor; CBD: cannabidiol; THC: tetrahydrocannabinol; BD: bis in die; NMDA: N-methyl-D-aspartate; MAO: monoamine oxidase; A*β*: amyloid beta.

## Data Availability

No personal data was collected from the interviewees and therefore no such data is kept or shared in any form.
